# Dynamic reward-penalty incentives and occupational health governance: an evolutionary game analysis with public health implications

**DOI:** 10.3389/fpubh.2026.1808355

**Published:** 2026-04-22

**Authors:** Yanan Li, Debin Fang, Tong Lou, Luping Jiang

**Affiliations:** 1 Research Center for Complexity Science and Management, Wuhan University, Wuhan, Hubei, China; 2 State Key Laboratory of Water Resources Engineering and Management, Wuhan University, Wuhan, Hubei, China; 3 South-Central Minzu University, Wuhan, Hubei, China; 4 School of Marine Engineering, Jimei University, Xiamen, Fujian, China; 5 School of Economics and Foreign Languages, Chengdu Technological University, Chengdu, Sichuan, China

**Keywords:** dynamic reward-penalty incentive, environmental regualtion, evolutionary game, occupational health and safety, occupational health governance, public health implications

## Abstract

**Introduction:**

Occupational health risks remain a significant public health concern in environmentally intensive industries, where inadequate regulatory enforcement and weak enterprise compliance can result in persistent workplace health hazards and potentially preventable long-term health burdens for workers. Understanding how environmental regulatory incentives influence enterprise occupational health management is essential for improving occupational health governance and generating public-health-relevant benefits.

**Methods:**

This study developed an evolutionary game framework to examine the strategic interactions between local regulators and enterprises under environmental regulation, with a focus on occupational health governance. By comparing static and state-dependent reward-penalty incentive structures, the model explored how different regulatory designs shaped enterprises' investment in occupational health protection over time under imperfect monitoring.

**Results:**

The analysis showed that static reward-penalty schemes were insufficient to sustain long-term active occupational health governance. In contrast, dynamically adjusted penalty mechanisms were more effective in promoting proactive enterprise investment in occupational health management and stabilizing long-term occupational health governance. The findings further indicate that enterprise behavioral responses are highly sensitive to not only the structure and intensity of regulatory incentives, but also the monitoring probability that passive governance can be detected under strict supervision.

**Discussion:**

These results highlight the importance of incentive design in linking environmental regulation with occupational health governance under imperfect monitoring. By demonstrating how regulatory incentives can improve occupational health governance and strengthen worker health protection in high-risk industrial settings, this study provides policy-relevant insights with broader public health relevance.

## 1 Introduction

Protecting occupational health in high-pollution industries is an integral component of public health protection, high-quality development, ecological sustainability, and human-centered modernization (1, 2). Industrial workers are often the primary recipients of risks generated in production processes, and their health conditions reflect not only environmental burdens but also the effectiveness of occupational health governance (3, 4). Environmental degradation and occupational health risks therefore arise simultaneously and are co-produced through industrial operations, with occupational health risks potentially contributing to broader long-term public health burdens (5). However, firms often invest insufficiently in occupational health protection, resulting in preventable health risks for workers (6–8). Information on internal health risks is also difficult for external regulators to observe, which weakens monitoring and enforcement, and makes regulatory judgments depend on imperfect but policy-relevant observable signals (9–11). Workers generally have limited bargaining power over firms' safety-related decisions, and regulators face constraints in detecting and verifying internal risk management practices (12). These conditions allow firms to shift risks inward rather than fully internalize them. Understanding how occupational health strategies emerge and evolve under regulatory pressure is therefore essential for advancing integrated environmental and health governance with public health prevention relevance.

In recent years, dynamic regulation has been promoted as a response to such governance challenges in both environmental protection and occupational health prevention (13–15). It has improved several environmental outcomes (16, 17). However, whether dynamic regulatory signals designed for environmental objectives also lead to sustained improvements in occupational health outcomes is uncertain. Although workplace hazards and environmental emissions originate in the same production processes, policy frameworks and academic research often treat them as separate governance domains (18). Firms may respond to visible environmental indicators that are closely monitored and publicly disclosed, while internal health protection is underprovided (19). This disconnect indicates that improved environmental regulation does not necessarily lead to better occupational health governance. This linkage depends on whether occupational health concerns are incorporated into broader compliance architectures, such as inspections, evaluations, or disclosure arrangements (20). This highlights the need to examine how dynamic regulatory incentives may affect occupational health governance through these institutional and behavioral channels.

Addressing this issue requires attention to how firms adjust their behavior over time. Compliance decisions related to occupational health protection are not static. They evolve through learning, imitation, expectation updating, and repeated interactions with regulators. Yet much of the existing literature treats compliance as a one-shot or static choice and does not explicitly model these adaptive processes (21, 22). As a result, there is limited understanding of how sequential rewards and penalties shape long-term investment in occupational health protection as an evolving governance strategy with public-health-relevant implications (23). Evolutionary game theory provides a useful analytical framework for examining such dynamics (24, 25). It allows for bounded rationality, strategic adaptation, and learning under uncertainty (26). However, regulatory incentives are generally modeled as fixed parameters, rather than as state-dependent instruments whose intensity may be adjusted in response to changing compliance conditions (27, 28). These limitations reduce the explanatory power of current models and constrain their policy relevance, which suggests the need for a framework that integrates adaptive behavioral responses with state-dependent reward–penalty adjustment. In the present study, however, the dynamic aspect of regulation is examined in a narrower sense, namely as the state-dependent adjustment of reward and penalty intensity within an evolutionary interaction rather than the full institutional complexity of adaptive governance (29).

To address these gaps, this study develops an evolutionary game model between local governments and high-pollution enterprises to examine occupational health governance as a strategic outcome of government-enterprise interaction, with public-health-relevant implications. By explicitly introducing occupational health risk into the environmental regulatory game, the study treats occupational health governance as more than a narrow compliance issue while avoiding the assumption that the model directly captures explicit epidemiological outcomes. The dynamic aspect of regulation is operationalized specifically through state-dependent reward-penalty adjustment within an evolutionary interaction. The model examines the conditions under which state-dependent reward–penalty mechanisms outperform static regulatory arrangements in sustaining occupational health investment. Numerical simulations are used to explore how benefit differentials, cost structures, reward intensity, penalty severity, and monitoring probability affect strategic evolution and equilibrium outcomes. Through these analyses, the study aims to aims to clarify the linkage between environmental regulation and occupational health governance and to offer insights into incentive-compatible regulatory design under imperfect monitoring.

The study is structured as follows. *Section 1* displays the introduction. *Section 2* reviews the related literature. *Section 3* presents the model assumptions and methodology. *Section 4* analyzes the evolutionary equilibria under alternative regulatory configurations, and *Section 5* reports numerical simulation results based on Matlab. *Section 6* discusses the main findings, and *Section 7* summarizes the conclusions and policy implications.

## 2 Literature review

Environmental regulation has long been recognized as a critical policy instrument for addressing industrial externalities and mitigating pollution-related social costs, including population-level health impacts (30–32). Early research primarily focused on command-and-control regulation and market-based instruments, including emission standards, pollution taxes, and tradable permits (33–35). Recent studies have shifted toward adaptive and performance-based regulatory frameworks (89), with increasing attention to how regulatory intensity, rewards, and sanctions may be adjusted in response to observed compliance conditions (14, 36, 37, 89). In this study, this dynamic aspect is examined specifically as state-dependent reward-penalty adjustment within a government-enterprise interaction. These mechanisms differ from traditional static regulation by allowing incentive intensity to vary with compliance performance rather than fully fixed. Empirical evidence supports the effectiveness of dynamic mechanisms in improving pollution control outcomes (38), stimulating green technological innovation (39, 40), and fostering long-term environmental improvements, particularly in developing economies where both regulatory capacity and firm behavior are still evolving (41, 42).

Despite these advances, much of the literature on environmental regulation remains focused on external environmental outcomes while occupational health outcomes, as a component of public health protection, are often overlooked. Even when integrated governance models such as ESG performance assessments are considered, occupational health tends to be treated as a marginal issue, rather than an integral component of overall regulatory success (43, 44). This gap in the literature is noteworthy. Environmental emissions and occupational health risks are co-produced within the same industrial processes, yet they differ in terms of visibility, temporal payoff structures, and regulatory salience (45). Environmental performance is often subject to continuous monitoring and public disclosure, while occupational health risks remain less visible and integrated into external compliance and supervisory systems to a lesser extent. As a result, firms may prioritize compliance with environmental regulations while continuing to neglect occupational health protections. Importantly, such a spillover is not automatic. Environmental regulation is more likely to influence occupational health governance when occupational health indicators are embedded in compliance evaluation, regulatory inspections, disclosure arrangements, or related supervisory processes (29, 46). Thus, there remains an urgent need for a framework that integrates occupational health alongside environmental performance, and clarifies the channels through which regulatory interventions may affect both domains.

Research on occupational health governance highlights persistent risks in high-pollution industries and their implications for worker health and related public health burdens (47–49). These studies emphasize the negative effects of workplace hazards, such as toxic exposure, dust inhalation, and unsafe working conditions, on productivity, health, and social welfare, particularly through cumulative exposure and delayed disease outcomes (49–51). Institutional analyses explore the roles of legal enforcement, supervision systems, and enterprise-level responsibility mechanisms in shaping occupational health outcomes (52, 53). However, much of the literature on occupational health remains static, with limited attention to how preventive governance evolves under changing regulatory conditions. Many studies focus on regulatory gaps, compliance failures, and fragmented governance structures (54–56). While these analyses are valuable for identifying governance deficiencies, they offer limited insight into how firms' occupational health governance strategies evolve over time in response to changing policy signals. Quantitative modeling in this area is also somewhat limited. Most studies rely on cost-benefit analysis or simple strategic models that assume fixed incentives and one-time decision-making (57, 58). These models often fail to capture critical aspects of occupational health governance, such as delayed health effects, probabilistic accidents, and firms' bounded rationality in decision-making. As a result, the literature offers limited understanding of how firms adapt their occupational health strategies in response to evolving regulatory pressures. This highlights the need for models that can capture adaptive behavior and learning processes in occupational health governance.

Evolutionary game theory has gained popularity in studies of environmental regulation and risk governance, but its application to occupational health governance remains limited (59–61). Existing applications of evolutionary game theory cover a broad range of topics, including emissions control, green technology diffusion, carbon mitigation, and environmental compliance (62, 63). These studies show how feedback effects, payoff differentials, and expectation updates shape equilibrium outcomes and system stability. Evolutionary games, therefore, offer a valuable framework for examining how occupational health governance evolves under state-dependent reward-penalty regulation (15).

However, several limitations can still be identified in the current literature, particularly regarding the modeling of occupational health governance under incomplete observability and delayed returns. First of all, most models simplify governance structures into two-party interactions, while giving limited attention to asymmetric information, delayed effects, and uneven cost-benefit exposure. These elements are crucial for a more complete understanding of occupational health risks. In addition, regulatory instruments are usually treated as static and exogenous parameters, which means that the adaptive adjustment of reward and penalty intensity is not always well-represented. Moreover, while some applications take government-enterprise interactions into consideration, few studies explicitly include occupational health governance within the context of evolutionary games. These gaps suggest that although evolutionary game theory provides a promising approach, it has not yet fully captured the complexity of co-governance that involves both environmental and occupational health risks.

Taken together, the existing literature offers valuable insights into environmental regulation, occupational health governance, and government-enterprise interaction. Nevertheless, these research streams leave several questions insufficiently explored. Dynamic incentive-penalty mechanisms have been shown to improve environmental performance although, their implications for occupational health governance remain insufficiently understood. Similarly, studies of occupational health governance have provided important institutional and descriptive analyses, yet they often rely on static perspectives that do not fully account for adaptive firm behavior under evolving regulatory conditions. As a result, the mechanisms through which firms adjust their occupational health strategies in response to changing incentives, enforcement signals, and observable compliance conditions remain only partially explained. Evolutionary game models offer tools that are well suited to address such dynamic processes. However, many existing applications employ static regulatory parameters or simplified interaction structures, which may limit their ability to capture the adaptive nature of real regulatory systems. In addition, the institutional linkage between environmental regulation and occupational health governance, as well as the role of imperfect observability in shaping regulatory effectiveness, has received relatively limited attention.

To address this need, the present study seeks to contribute to the literature by developing an evolutionary game framework that incorporates occupational health governance into the analysis of environmental regulation. More specifically, the study focuses on state-dependent reward-penalty adjustment within a government-enterprise interaction setting, and examines how alternative regulatory configurations affect the long-term evolution of occupational health strategies, with implications for worker protection and related public health concerns.

## 3 Methods

### 3.1 Conceptual transmission mechanism

**Figure 1:**
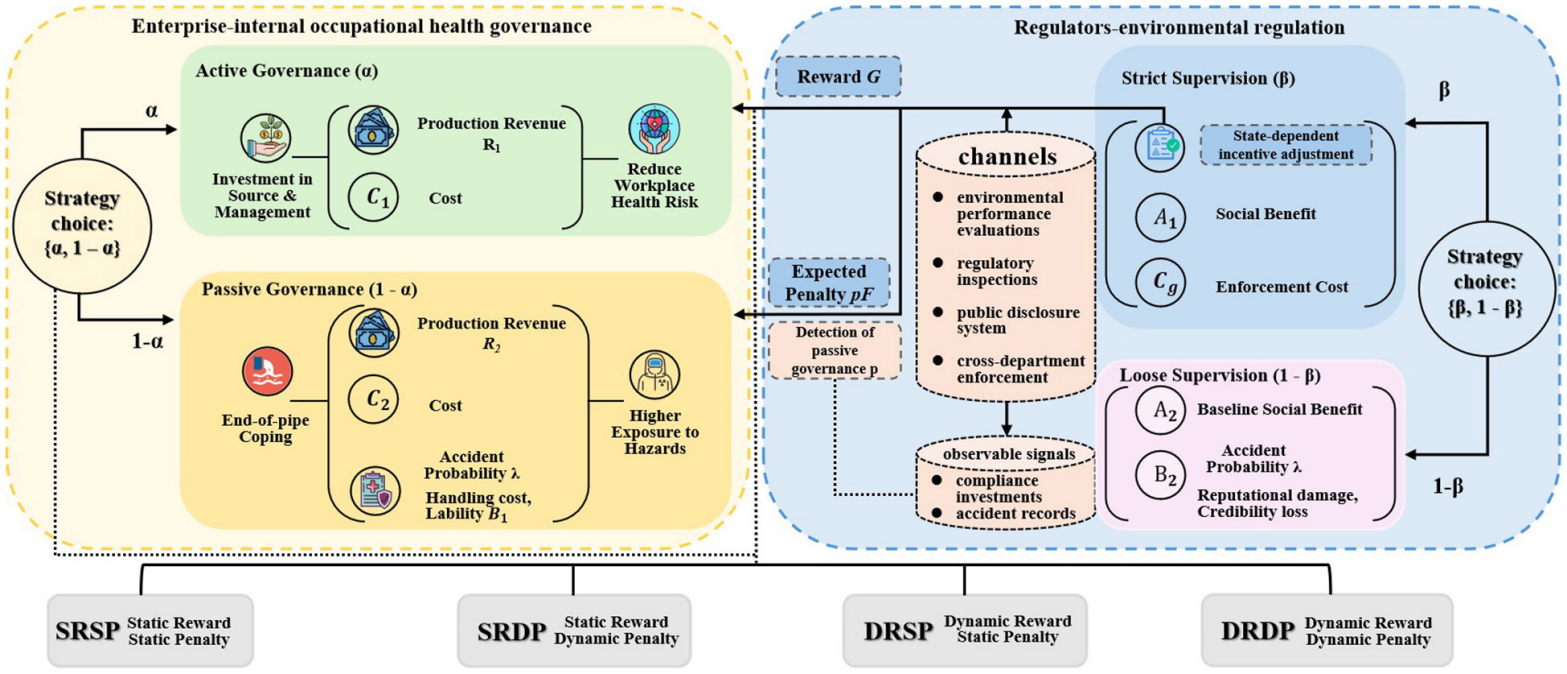
Transmission mechanism linking environmental regulation and occupational health governance.

Building on the literature reviewed above, Figure 1 presents the conceptual institutional transmission mechanism through which environmental regulation may influence enterprise occupational health governance.

As illustrated in Figure 1, environmental regulation is not assumed to improve occupational health protection automatically. Instead, its influence is transmitted through institutionally embedded channels, such as regulatory inspections, performance evaluations, disclosure arrangements, and observable compliance signals. Through these channels, external regulatory pressure is translated into observable compliance signals, which in turn shape firms' occupational health strategies and affect workplace health risks.

### 3.2 Fundamental assumptions

This section formalizes the evolutionary interaction between local governments and high-pollution enterprises under alternative regulatory conditions. In this study, occupational health protection is treated as an enterprise governance outcome with public-health relevant implications. Occupational health risks are generated inside production processes and are only partially observable to external regulators. Accordingly, regulatory design is modeled as a mechanism that shapes enterprise incentives and learning dynamics under imperfect monitoring rather than full observability (64).

To explore how firms internalize policy signals and adjust their occupational health strategies, a dynamic reward-penalty evolutionary game model is constructed. In particular, strict supervision does not imply that regulators can perfectly identify firms' true internal behavior. Instead, it increases the probability that passive governance can be detected through observable compliance signals, such as inspection records, compliance investments, occupational health documentation, or accident-related evidence.

To evaluate how different governance designs shape behavioral evolution, we introduce four regulatory configurations that vary in the static or dynamic nature of their reward and penalty components: static reward and static penalty (SRSP), static reward and dynamic penalty (SRDP), dynamic reward and static penalty (DRSP), and dynamic reward and dynamic penalty (DRDP). Comparing these scenarios allows us to assess how changes in regulatory responsiveness influence equilibrium stability, strategic convergence, and policy effectiveness. The next subsection specifies assumptions, payoff structures, and state-dependent incentive adjustment rules underpinning the model.

**Hypothesis 1:** The game participants are two parties. The primary corporate entity responsible for providing occupational health services as a social responsibility is *Participant 1*, while government regulatory authorities are *Participant 2*. As the direct implementers of regulation operating under the dual constraints of fiscal budgets and performance assessments, local governments are designated as the primary strategic players in this game. Both parties exhibit information asymmetry and bounded rationality. The interaction is modeled as a state-dependent incentive adjustment where the Regulatory Authority seeks to maximize social welfare net of enforcement costs, including the prevention of occupational health risks and associated public health burdens, while the Corporate Agent aims to minimize the sum of abatement costs and expected fines.

**Hypothesis 2:** When the government opts for strict regulation, enterprises may implement active governance strategy by increasing investment in occupational health management systems, aimed at reducing workplace health risks and preventing occupational diseases. This ensures employees receive standard-compliant health protection that meets regulatory requirements. Alternatively, enterprises may refrain from active governance or even adopt a passive governance approach, resulting in higher exposure to occupational health hazards. Let α denote the probability that regulated entities adopt an active governance strategy, then the probability of adopting a passive approach is 1–α. Faced with these two distinct corporate choices, regulatory authority may opt for strict supervision, under which occupational health performance is assessed through inspections and observable compliance signals. Alternatively, they may choose loose supervision, in which case no rewards or penalties are applied. Let β denote the probability that local governments choose strict supervision, then the probability of choosing loose supervision is 1–β. Here, 0 ≤ α, β ≤ 1. Therefore, the strategy space for the regulated firms is defined as {α, 1–α} = {Active Governance, Passive Governance}. The local regulators face a binary choice between {β, 1–β} = {Strict Supervision, Loose Supervision}.

**Hypothesis 3:** In the evolutionary game, the costs associated with enterprises proactively adopting the active governance strategy and the passive governance strategy are *C*_1_ and *C*_2_, respectively. Since proactively active governance requires substantial initial investments in source control technologies, specialized personnel, and comprehensive management systems, we assume *C*_1_ > *C*_2_ (58, 65). The baseline production revenue for enterprises proactively implementing active governance is denoted as *R*_1_. When government regulators adopt a strict supervision strategy, it provides incentive support *G*, such as fiscal subsidies or tax breaks, to enterprises that successfully implement active governance, thereby encouraging preventive investment in occupational health protection. Furthermore, strict regulatory enforcement enhances social welfare implications. Let *A*_1_ represent the social benefit generated under strict supervision when firms comply, whereas *A*_2_ represents the baseline social benefit under loose supervision. Since strict supervision more effectively mitigates systemic risks and improves long-term social welfare compared to a laissez-faire approach, we assume 0 < A_2_ < A_1_.

**Hypothesis 4:** When an enterprise opts for passive governance (i.e., relying on end-of-pipe coping rather than proactive prevention), its production revenue is denoted as *R*_2_. Due to the reduction in safety expenditures, such enterprises typically secure higher short-term yields, implying *R*_2_ > *R*_1_ (66). While safety incidents can occur under any regime, empirical evidence suggests the probability is significantly heightened when firms neglect source control. For model simplification, this study focuses specifically on the accident probability λ associated with passive governance (67, 68). In the event of an accident, the enterprise incurs direct handling costs and liability losses, reflecting both immediate safety incidents and longer-term occupational health consequences, denoted by *B*_1_. Simultaneously, the regulatory agency suffers reputational damage and credibility loss, denoted by *B*_2_. Crucially, passive governance is not perfectly observable. Instead, regulators can identify passive governance with probability *p*, where 0 < *p*<*1*. The parameter *p* represents monitoring effectiveness or inspection accuracy based on observable signals such as compliance records, occupational health documentation, third-party verification, or accident-related evidence. Accordingly, when passive governance is detected under strict supervision, the government imposes a penalty *F*, and the expected penalty becomes *pF*.

**Hypothesis 5:** Implementing strict supervision incurs a direct regulatory enforcement cost *C*_*g*_ (e.g., expenditures on inspection personnel, monitoring equipment, and administrative processing). Conversely, when regulators opt for loose supervision, they avoid these enforcement costs but do not generate effective monitoring signals for identifying passive governance. Consequently, under loose supervision, the government refrains from dispensing rewards (*G*) or imposing penalties (*F*). The parameter settings are as shown in Table 1.

**Table 1:** Descriptions of parameters.

Parameters	Descriptions	Notes
*C*_1_	Production costs associated with enterprises adopting the active governance strategy.	*C*_1_ > 0
*C*_2_	Production costs associated with enterprises adopting the passive governance strategy.	0 < *C*_2_ < *C*_1_
*C*_*g*_	Costs associated with local government regulators implementing strict supervision.	*C*_*g*_ > 0
*R*_1_	The baseline production revenue for enterprises implementing active governance.	*R*_1_ > 0
*R*_2_	The baseline production revenue for enterprises implementing passive governance.	0 < *R*_1_ < *R*_2_
*A*_1_	The social benefit generated under strict supervision.	*A*_1_ > 0
*A*_2_	The social benefit generated under loose supervision.	0 < *A*_2_ < *A*_1_
*G*	The incentive rewards when government regulators adopt a strict supervision strategy to enterprises that successfully implement active governance.	*G* > 0
*F*	The penalties when government regulators adopt a strict supervision strategy to enterprises with non-compliance.	*F* > 0
λ	The accident probability associated with passive governance.	0 < λ ≤ 1
*B*_1_	The losses incurred from occupational health and safety incidents caused by enterprises implementing passive governance.	*B*_1_ > 0
*B*_2_	The losses to government performance and credibility resulting from occupational health and safety incidents.	*B*_2_ > 0
*p*	Probability that passive governance is correctly detected under strict supervision.	0 < *p* < 1

### 3.3 Research methods

#### 3.3.1 Evolutionary game payoff matrix

Based on the established assumptions, the strategic interaction between local governments and high-pollution enterprises is formalized as a two-player evolutionary game model. Bounded rationality is assumed for both participants; consequently, strategic updates are driven by limited learning, imitation, and payoff comparisons rather than perfect optimization. The strategy space for enterprises is defined as {Active Governance (α), Passive Governance (1–α)}. Conversely, the strategy space for local governments is defined as {Strict Supervision (β), Loose Supervision (1–β)}. The payoff functions are constructed to reflect direct production costs, delayed social benefits, regulatory risks, and the public health implications of occupational health risk exposure. The resulting payoff matrix is presented in Table 2.

**Table 2:** Payoff matrix for the local government and high-pollution enterprises.

Strategy selection		Local government	
		Strict supervision (β)	Loose supervision (1–β)
High-pollution enterprises	Active governance (α)	*R*_1_ + *G* − *C*_1_, *A*_1_ − *G* − *C*_*g*_	*R*_1_ − *C*_1_, *A*_2_
	Passive governance (1–α)	*R*_2_ − *pF* − *C*_2_ − λ*B*_1_, *A*_1_ − *C*_*g*_ + *pF* − λ*B*_2_	*R*_2_ − *C*_2_ − λ*B*_1_, *A*_2_ − λ*B*_2_

#### 3.3.2 Replication dynamic equations and equilibrium points

To capture the adaptive evolution of these strategies, replicator dynamics are introduced as the core behavioral adjustment mechanism. The replicator dynamic function expresses how the proportion of actors adopting a given strategy changes over time, capturing how preventive occupational health behaviors diffuse under regulatory pressure. When the expected payoff of a strategy is higher than the average payoff in the population, its proportion increases; conversely, it declines when its payoff is inferior. The resulting payoff matrix enables derivation of the replicator dynamic equations for enterprises and governments, respectively (8, 25).

The expected returns of high-pollution enterprises choosing active governance and passive governance strategies are *E*_11_ and *E*_12_, respectively, while the average expected return is *E*_1_, as expressed in Equations 1–3, respectively.


$$\begin{array}{l} E_{11}=\beta \left(R_{1}+G-C_{1}\right)+\left(1-\beta \right)\left(R_{1}-C_{1}\right) \end{array}$$ (1)

$$\begin{array}{l} E_{12}=\beta \left(R_{2}-pF-C_{2}-\lambda B_{1}\right)+\left(1-\beta \right)\left(R_{2}-C_{2}-\lambda B_{1}\right) \end{array}$$ (2)

$$\begin{array}{l} E_{1}=\alpha E_{11}+\left(1-\alpha \right)E_{12} \end{array}$$ (3)

Consequently, the replicated dynamic equation of the enterprise's strategy evolution is shown in Equation 4.


$$\begin{array}{l} F\left(\alpha \right)=d\alpha /dt=\alpha \left(E_{11}-E_{1}\right) \end{array}$$

$$\begin{array}{l} =\alpha \left(1-\alpha \right)\left[C_{2}+R_{1}-R_{2}-C_{1}+\lambda B_{1}+\beta \left(G+pF\right)\right] \end{array}$$ (4)

Similarly, the expected returns of local government choosing strict supervision and loose supervision strategies are E_21_ and E_22_, respectively, while the average expected return is E_2_, as expressed in Equations 5–7, respectively.


$$\begin{array}{l} E_{21}=\alpha \left(A_{1}-G-C_{g}\right)+\left(1-\alpha \right)\left(A_{1}-C_{g}+pF-\lambda B_{2}\right) \end{array}$$ (5)

$$\begin{array}{l} E_{22}=\alpha A_{2}+\left(1-\alpha \right)\left(A_{2}-\lambda B_{2}\right) \end{array}$$ (6)

$$\begin{array}{l} E_{2}=\beta E_{21}+\left(1-\beta \right)E_{22} \end{array}$$ (7)

The replicated dynamic equations of the government's behavioral strategy evolution is derived in Equation 8.


$$\begin{array}{l} F\left(\beta \right)=d\beta /dt=\beta \left(E_{21}-E_{2}\right) \end{array}$$

$$\begin{array}{l} =\beta \left(1-\beta \right)\left[A_{1}-C_{g}+pF-A_{2}-\alpha \left(G+pF\right)\right] \end{array}$$ (8)

According to the stability theorem of differential equations, the system's equilibrium points are identified by setting the time derivatives equal to zero [*F*(α) = 0] and [*F*(β) = 0]. This yields five local equilibrium points: (0, 0), (1, 0), (0, 1), (1, 1), and the internal saddle point (α^*^, β^*^), which is defined in Equations 9, 10, respectively.


$$\begin{array}{l} \alpha^{*}=\frac{A_{1}-C_{g}+pF-A_{2}}{G+pF} \end{array}$$ (9)

$$\begin{array}{l} \beta^{*}=\frac{C_{1}+R_{2}-R_{1}-C_{2}-\lambda B_{1}}{G+pF} \end{array}$$ (10)

Overall, the modeling framework is designed to examine occupational health protection as a dynamic, public-health-relevant process shaped by regulatory incentives rather than a static compliance choice.

## 4 Analysis of evolutionary stability strategy of occupational health governance

Based on the replicator dynamic equations derived in *Section 3*, this section employs the Lyapunov stability method to systematically analyze the asymptotic stability of equilibrium points under four regulatory configurations, with a focus on the sustainability of occupational health protection outcomes. This analysis identifies the boundary conditions required for the system to converge to the ideal state of Active Governance and Strict Supervision, which corresponds to sustained occupational health protection under effective regulatory oversight.

### 4.1 Strategy stability analysis under static reward and static penalty (SRSP)

In the SRSP mechanism, both the reward (*G*) and penalty (*pF*) are exogenous constants that do not adjust to changes in corporate occupational health protection rates. To evaluate stability, we calculate the Jacobian matrix (*J*) of the system as defined in Equation 11.


$$\begin{array}{l} J=\left[\begin{array}{cc} \frac{\partial F\left(\alpha \right)}{\partial \alpha } & \frac{\partial F\left(\alpha \right)}{\partial \beta }\\ \frac{\partial F\left(\beta \right)}{\partial \alpha } & \frac{\partial F\left(\beta \right)}{\partial \beta } \end{array}\right]=\left[\begin{array}{cc} a_{11} & a_{12}\\ a_{21} & a_{22} \end{array}\right] \end{array}$$ (11)

where the partial derivatives corresponding to the SRSP scenario are derived in Equations 12–15, respectively.


$$\begin{array}{l} a_{11}=\left(1-2\alpha \right)\left[C_{2}+R_{1}-R_{2}-C_{1}+\lambda B_{1}+\beta \left(G+pF\right)\right] \end{array}$$ (12)

$$\begin{array}{l} a_{12}=\alpha \left(1-\alpha \right)\left(G+pF\right) \end{array}$$ (13)

$$\begin{array}{l} a_{21}=\beta \left(1-\beta \right)\left(-G-pF\right) \end{array}$$ (14)

$$\begin{array}{l} a_{22}=\left(1-2\beta \right)\left[A_{1}-C_{g}+pF-A_{2}-\alpha \left(G+pF\right)\right] \end{array}$$ (15)

According to Friedman's theory, an equilibrium point is an Evolutionarily Stable Strategy (ESS) if it satisfies both the determinant condition [*det(J)* > 0] and the trace condition [*tr(J)* < 0], which is shown in Equations 16, 17, respectively.


$$\begin{array}{l} \det\left(J\right)=\left|\begin{array}{cc} a_{11} & a_{12}\\ a_{21} & a_{22} \end{array}\right|=a_{11}a_{22}-a_{12}a_{21}>0 \end{array}$$ (16)

$$\begin{array}{l} tr\left(J\right)=a_{11}+a_{22}<0 \end{array}$$ (17)

The local stability analysis for the five equilibrium points is summarized in Table 3.

**Table 3:** Local stability analysis of equilibrium points under SRSP.

Equilibrium	det(J)	+/−	tr(*J*)	+/−
(0, 0)	(*C*_2_ + *R*_1_ − *R*_2_ − *C*_1_ + λ*B*_1_)(*A*_1_ − *C*_*g*_ + *pF* − *A*_2_)	−	*C*_2_ + *R*_1_ − *R*_2_ − *C*_1_ + λ*B*_1_ + *A*_1_ − *C*_*g*_ + *pF* − *A*_2_	N
(0, 1)	−(*C*_2_ + *R*_1_ − *R*_2_ − *C*_1_ + λ*B*_1_ + *G* + *pF*)(*A*_1_ − *C*_*g*_ + *pF* − *A*_2_)	−	*C*_2_ + *R*_1_ − *R*_2_ − *C*_1_ + λ*B*_1_ + *G* + *pF*−(*A*_1_ − *C*_*g*_ + *pF* − *A*_2_)	N
(1, 0)	−(*C*_2_ + *R*_1_ − *R*_2_ − *C*_1_ + λ*B*_1_)(*A*_1_ − *C*_*g*_ − *G* − *A*_2_)	−	−(*C*_2_ + *R*_1_ − *R*_2_ − *C*_1_ + λ*B*_1_) + (*A*_1_ − *C*_*g*_ − *G* − *A*_2_)	N
(1, 1)	(*C*_2_ + *R*_1_ − *R*_2_ − *C*_1_ + λ*B*_1_ + *G* + *pF*)(*A*_1_ − *C*_*g*_ − *G* − *A*_2_)	−	−(*C*_2_ + *R*_1_ − *R*_2_ − *C*_1_ + λ*B*_1_ + *G* + *pF*)−(*A*_1_ − *C*_*g*_ − *G* − *A*_2_)	N
(α^*^, β^*^)	*M*	+	0	0

It is important to note the following conditions. When firms implement passive governance, the government's net payoff from strict supervision (i.e., social benefits minus regulatory costs plus the expected penalty revenues) exceeds that from loose supervision, that is, *A*_1_-*C*_g_+*pF*–*A*_2_ > 0. When firms proactively adopt the active governance strategy, the government's net payoff under strict supervision is lower than that under loose supervision, namely *A*_1_-*C*_g_-*G*–*A*_2_ < 0. When the government adopts loose supervision, the firms' payoff from active governance is lower than from passive governance, expressed as *C*_2_+*R*_1_-*R*_2_-*C*_1_+λ*B*_1_ < 0. Conversely, under strict supervision, the firms' payoff from active governance is higher than that from passive governance, that is, *C*_2_+*R*_1_-*R*_2_-*C*_1_+λ*B*_1_+*G*+*pF* > 0. This highlights that supervision effectiveness depends not only on the statutory penalty level but also on the probability of detecting passive governance.

As indicated in Table 3, none of the five equilibrium points satisfy the ESS criteria under SRSP, indicating that static regulation fails to sustain stable occupational health protection. The determinant of the Jacobian matrix at the internal point (α^*^, β^*^) is positive, but its trace is zero [*tr(J)* = 0]. This characteristic indicates that the system is a conservative system with closed orbits, meaning the strategies of governments and enterprises will exhibit periodic oscillations, implying fluctuating levels of occupational health protection rather than long-term prevention.

### 4.2 Strategy stability analysis under static reward and dynamic penalty (SRDP)

In the SRDP scenario, we introduce a dynamic penalty mechanism where the fine intensity is negatively correlated with the industry's overall compliance level. Because passive governance is only imperfectly observable, the effective penalty under strict supervision is modeled as an expected dynamic penalty, defined as *F*(α) = *p*(1–α)*F*_*m*_, where *F*_*m*_ represents the maximum penalty and *p* reflects monitoring effectiveness. While the reward *G* is treated as a constant. The replicator dynamic equation for the enterprise is given by Equation 18.


$$\begin{array}{l} F\left(\alpha \right)=d\alpha /dt=\alpha \left(E_{11}-E_{1}\right)= \end{array}$$

$$\begin{array}{l} \alpha \left(1-\alpha \right)\left[C_{2}+R_{1}-R_{2}-C_{1}+\lambda B_{1}+\beta \left(G+p\left(1-\alpha \right)F_{m}\right)\right] \end{array}$$ (18)

The replicator dynamic equation for the government is given by Equation 19.


$$\begin{array}{l} f(\beta )=d\beta /dt=\beta (E_{21}-E_{2})\\ \;=\beta (1-\beta )[A_{1}-C_{g}+p(1-\alpha )F_{m}-A_{2}-\\ \;\alpha (G+p(1-\alpha )F_{m})] \end{array}$$ (19)

Under SRDP, the firms' replicator dynamic equation shows that the growth rate of the probability that firms adopt active governance is positively associated with the payoff difference between active and passive governance (*R*_1_-*R*_2_), the government reward (*G*), and the expected penalty *p*(1–α)*F*_*m*_. In contrast, it is negatively associated with the cost difference between active and passive governance (*C*_1_-*C*_2_). Similarly, the government's replicator dynamic equation indicates that the growth rate of adopting a strict supervision strategy is negatively related to the reward (*G*) granted to firms that implement active governance, while it is positively related to the expected penalty (1–α)*F*_*m*_, which depends jointly on statutory intensity and monitoring effectiveness.

Substituting this into the replicator dynamic equations, we analyze the stability of the equilibrium points. Five local equilibrium points can be obtained: (0, 0), (1, 0), (0, 1), (1, 1) and *M*(α^*^,β^*^), where

$\alpha^{*}=\frac{G+2pF_{m}-\sqrt{G^{2}-4pF_{m}\left(A_{1}-C_{g}-A_{2}-G\right)}}{2pF_{m}}$, $\beta^{*}=\frac{2\left(C_{1}+R_{2}-R_{1}-C_{2}-\lambda B_{1}\right)}{G+\sqrt{G^{2}-4pF_{m}\left(A_{1}-C_{g}-A_{2}-G\right)}}$. The results are presented in Table 4.

**Table 4:** Local stability analysis of equilibrium points under SRDP.

Equilibrium	det(J)	+/−	tr (*J*)	+/−
(0, 0)	(*C*_2_ + *R*_1_ − *R*_2_ − *C*_1_+λ*B*_1_)(*A*_1_ − *C*_*g*_ + *pF* − *A*_2_)	−	*C*_2_ + *R*_1_ − *R*_2_ − *C*_1_+λ*B*_1_ + *A*_1_ − *C*_*g*_ + *pF* − *A*_2_	N
(0, 1)	−(*C*_2_ + *R*_1_ − *R*_2_ − *C*_1_+λ*B*_1_ + *G* + *pF*)(*A*_1_ − *C*_*g*_ + *pF* − *A*_2_)	−	*C*_2_ + *R*_1_ − *R*_2_ − *C*_1_+λ*B*_1_ + *G* + *pF*−(*A*_1_ − *C*_*g*_ + *pF* − *A*_2_)	N
(1, 0)	−(*C*_2_ + *R*_1_ − *R*_2_ − *C*_1_+λ*B*_1_)(*A*_1_ − *C*_*g*_ − *G* − *A*_2_)	−	−(*C*_2_ + *R*_1_ − *R*_2_ − *C*_1_+λ*B*_1_) + (*A*_1_ − *C*_*g*_ − *G* − *A*_2_)	N
(1, 1)	(*C*_2_ + *R*_1_ − *R*_2_ − *C*_1_+λ*B*_1_ + *G*)(*A*_1_ − *C*_*g*_ − *G* − *A*_2_)	−	−(*C*_2_ + *R*_1_ − *R*_2_ − *C*_1_+λ*B*_1_ + *G*)−(*A*_1_ − *C*_*g*_ − *G* − *A*_2_)	N
(α^*^, β^*^)	*M*	+		−

When the condition (*R*_1_ − *C*_1_ + *G* > *R*_2_ − *C*_2_ − λ*B*_1_ − *p*(1α)*F*_*m*_) is met, the system can converge to the mixed strategy equilibrium point *M*(α^*^, β^*^), representing a stable level of occupational health protection supported by adaptive penalty mechanisms. This suggests that linking penalties to compliance performance can still generate a stabilizing negative feedback loop, but its effectiveness depends on monitoring probability *p*. When *p* is low, dynamic penalties lose disciplinary strength even if the statutory ceiling *F*_*m*_ remains high.

### 4.3 Strategy stability analysis under dynamic reward and static penalty (DRSP)

In the DRSP scenario, the government employs a dynamic incentive strategy where the reward intensity is linked to the prevalence of active governance. Specifically, the dynamic reward is defined as *G*(α) = α*G*_*m*_, where *G*_*m*_ denotes the maximum reward granted to firms that adopt active governance under strict supervision. while the penalty remains a fixed statutory sanction. Because passive governance is not perfectly observable, the effective penalty under strict supervision is represented by its expected value *pF*. Accordingly, the replicator dynamic equation describing the evolutionary behavior of firms can be expressed in Equation 20.


$$\begin{array}{l} F\left(\alpha \right)=d\alpha /dt=\alpha \left(E_{11}-E_{1}\right) \end{array}$$

$$\begin{array}{l} =\alpha \left(1-\alpha \right)\left[C_{2}+R_{1}-R_{2}-C_{1}+\lambda B_{1}+\beta \left(\alpha G_{m}+pF\right)\right] \end{array}$$ (20)

The replicator dynamic equation for the government is given by Equation 21.


$$\begin{array}{l} F\left(\beta \right)=d\beta /dt=\beta \left(E_{21}-E_{2}\right) \end{array}$$

$$\begin{array}{l} =\beta \left(1-\beta \right)\left[A_{1}-C_{g}+pF-A_{2}-\alpha \left(\alpha G_{m}+pF\right)\right] \end{array}$$ (21)

Under DRSP, the firms' replicator dynamic equation shows that the probability of implementing active governance increases with the payoff difference between active and passive governance (*R*_1_−*R*_2_), the reward α*G*_*m*_, and the expected penalty (*pF*), but decreases with the corresponding cost difference (*C*_1_−*C*_2_). Similarly, the government's replicator dynamic equation further indicates that the likelihood of adopting strict supervision declines as the reward α*G*_*m*_, but rises with the expected penalty(*pF*).

Similarly, by setting *F*(α) = 0 and *F*(β) = 0, five local equilibrium points can be obtained: (0, 0), (1, 0), (0, 1), (1, 1) and (α^*^, β^*^), where $\alpha^{*}=\frac{-pF+\sqrt{p^{2}F^{2}-4G_{m}\left(A_{1}-C_{g}-A_{2}+pF\right)}}{2G_{m}}$, $\beta^{*}=\frac{2\left(C_{1}+R_{2}-R_{1}-C_{2}-\lambda B_{1}\right)}{pF+\sqrt{p^{2}F^{2}+4G_{m}\left(A_{1}-C_{g}-A_{2}+pF\right)}}$. The stability analysis for this configuration is summarized in Table 5.

**Table 5:** Local stability analysis of equilibrium points under DRSP.

Equilibrium	det (J)	+/−	tr(*J*)	+/−
(0, 0)	(*C*_2_ + *R*_1_ − *R*_2_ − *C*_1_+λ*B*_1_)(*A*_1_ − *C*_*g*_ + *pF* − *A*_2_)	−	*C*_2_ + *R*_1_ − *R*_2_ − *C*_1_+λ*B*_1_ + *A*_1_ − *C*_*g*_ + *pF* − *A*_2_	N
(0, 1)	−(*C*_2_ + *R*_1_ − *R*_2_ − *C*_1_+λ*B*_1_ + *pF*)(*A*_1_ − *C*_*g*_ + *pF* − *A*_2_)	−	*C*_2_ + *R*_1_ − *R*_2_ − *C*_1_+λ*B*_1_ + *pF*−(*A*_1_ − *C*_*g*_ + *pF* − *A*_2_)	N
(1, 0)	−(*C*_2_ + *R*_1_ − *R*_2_ − *C*_1_+λ*B*_1_)(*A*_1_ − *C*_*g*_ − *G* − *A*_2_)	−	−(*C*_2_ + *R*_1_ − *R*_2_ − *C*_1_+λ*B*_1_) + (*A*_1_ − *C*_*g*_ − *G* − *A*_2_)	N
(1, 1)	(*C*_2_ + *R*_1_ − *R*_2_ − *C*_1_+λ*B*_1_ + *G* + *pF*)(*A*_1_ − *C*_*g*_ − *G* − *A*_2_)	−	−(*C*_2_ + *R*_1_ − *R*_2_ − *C*_1_+λ*B*_1_ + *G* + *pF*)−(*A*_1_ − *C*_*g*_ − *G* − *A*_2_)	N
(α^*^, β^*^)	*M*	+		+

As indicated in Table 5, all five equilibrium points become unstable under DRSP, indicating that reward-driven regulation alone is insufficient to ensure stable occupational health outcomes. Similar to the static mechanism, the DRSP configuration often fails to eliminate cyclical fluctuations completely. While dynamic rewards can alter the frequency of oscillations, they are less effective than dynamic penalties in stabilizing the system.

### 4.4 Strategy stability analysis under dynamic reward and dynamic penalty (DRDP)

Finally, we analyze the fully dynamic DRDP mechanism, where both instruments are endogenous. Specifically, the dynamic reward is defined as (α) = α*G*_*m*_, where *G*_*m*_ denotes the maximum reward. Likewise, the dynamic penalty is specified as (α) = *p*(1−α)*F*_*m*_, where *F*_*m*_ represents the maximum penalty. This creates a dual-feedback mechanism. The replicator dynamic equation for firms can be expressed in Equation 22.


$$\begin{array}{l} F\left(\alpha \right)=d\alpha /dt=\alpha \left(E_{11}-E_{1}\right)=\\ \alpha \left(1-\alpha \right)\left[C_{2}+R_{1}-R_{2}-C_{1}+\lambda B_{1}+\beta \left(\alpha G_{m}+p\left(1-\alpha \right)F_{m}\right)\right] \end{array}$$ (22)

The replicator dynamic equation for the government is given by Equation 23.


$$\begin{array}{l} F\left(\beta \right)=d\beta /dt=\beta \left(E_{21}-E_{2}\right)=\\ \beta \left(1-\beta \right)\left[A_{1}-C_{g}+p\left(1-\alpha \right)F_{m}-A_{2}-\alpha \left(\alpha G_{m}+p\left(1-\alpha \right)F_{m}\right)\right] \end{array}$$ (23)

In the model with DRDP, the firms' replicator dynamic equation shows that the growth rate of the probability of implementing active governance increases with the payoff difference between active and passive governance (*R*_1_−*R*_2_), as well as with the reward (α*G*_*m*_) and the expected penalty *p*(1−α)*F*_*m*_. Conversely, it decreases with the cost differential (*C*_1_−*C*_2_).The government's replicator dynamic equation further indicates that the growth rate of adopting a strict supervision strategy declines as the reward (α*G*_*m*_), but rises with the expected penalty (1− α)*F*_*m*_.

Similarly, by setting *F*(α) = 0 and *F*(β) = 0, five local equilibrium points can be obtained: (0, 0), (1, 0), (0, 1), (1, 1) and (α^*^, β^*^), where $\alpha^{*}=\frac{pF-\sqrt{p^{2}F^{2}-\left(pF-G\right)\left(A_{1}-C_{g}-A_{2}+pF\right)}}{pF-G},$

$\beta^{*}=\frac{C_{1}+R_{2}-R_{1}-C_{2}-\lambda B_{1}}{\sqrt{p^{2}F^{2}-\left(pF-G\right)\left(A_{1}-C_{g}-A_{2}+pF\right)}}$. The local stability properties of these equilibrium points are summarized in Table 6.

**Table 6:** Local stability analysis of equilibrium points under DRDP.

Equilibrium	det (*J*)	+/−	tr(*J*)	+/−
(0, 0)	(*C*_2_ + *R*_1_ − *R*_2_ − *C*_1_+λ*B*_1_)(*A*_1_ − *C*_*g*_ + *pF* − *A*_2_)	−	*C*_2_ + *R*_1_ − *R*_2_ − *C*_1_+λ*B*_1_ + *A*_1_ − *C*_*g*_ + *pF* − *A*_2_	N
(0, 1)	−(*C*_2_ + *R*_1_ − *R*_2_ − *C*_1_+λ*B*_1_ + *pF*)(*A*_1_ − *C*_*g*_ + *pF* − *A*_2_)	−	*C*_2_ + *R*_1_ − *R*_2_ − *C*_1_+λ*B*_1_ + *pF*−(*A*_1_ − *C*_*g*_ + *pF* − *A*_2_)	N
(1, 0)	−(*C*_2_ + *R*_1_ − *R*_2_ − *C*_1_+λ*B*_1_)(*A*_1_ − *C*_*g*_ − *G* − *A*_2_)	−	−(*C*_2_ + *R*_1_ − *R*_2_ − *C*_1_+λ*B*_1_) + (*A*_1_ − *C*_*g*_ − *G* − *A*_2_)	N
(1, 1)	(*C*_2_ + *R*_1_ − *R*_2_ − *C*_1_+λ*B*_1_ + *G*)(*A*_1_ − *C*_*g*_ − *G* − *A*_2_)	−	−(*C*_2_ + *R*_1_ − *R*_2_ − *C*_1_+λ*B*_1_ + *G*)−(*A*_1_ − *C*_*g*_ − *G* − *A*_2_)	N
(α^*^, β^*^)	*M*	+		−

As indicated in Table 6, the analysis confirms that DRDP offers the most robust stability characteristics, corresponding to the highest level of sustained occupational health protection. When the net payoff of active governance exceeds that of passive strategies *R*_1_−*C*_1_+α*G*_*m*_>*R*_2_−*C*_2_−λ*B*_1_−*p*(1−α)*F*_*m*_, the internal equilibrium point *M*(α^*^, β^*^), emerges as an asymptotically stable equilibrium.

A comparative analysis of the system's stability results across the four mechanisms yields several insights. First, DRDP remains the most robust configuration within the modeled setting. However, its stabilizing effect depends not only on dynamic adjustment of rewards and penalties, but also on the monitoring probability *p*. Second, high penalty ceilings *F*_*m*_ are more effective at shifting the equilibrium coordinate α^*^ (firm compliance rate) to the right than equivalent increases in reward ceilings (*G*_*m*_). Third, the dynamic adjustment naturally creates a “check and balance” that prevents fiscal exhaustion, as rewards decrease when compliance becomes the norm (high α), and enforcement costs drop when violations decrease.

## 5 Numerical simulation

To verify the theoretical stability analysis, numerical simulations are conducted using MATLAB. The dynamic reward-penalty mechanism was simulated under different regulatory mechanisms. This section analyzes how the system evolves from initial states to equilibrium. Additionally, the sensitivity of strategic choices to key parameters is examined.

### 5.1 Parameter assignment and baseline scenario

The simulation parameters are calibrated to satisfy the stability condition derived in the DRDP analysis: *R*_1_ − *C*_1_ + *G* > *R*_2_ − *C*_2_ − λ*B*_1_ − *pF*. This condition ensures that under maximum enforcement intensity, the net payoff of active governance exceeds that of passive governance, making convergence theoretically possible. The baseline parameter values are presented in Table 7.

**Table 7:** Initial parameter assignments for the two-party evolutionary game.

Parameter	*R*_1_	*R*_2_	*C*_1_	*C*_2_	*C*_*g*_	λ	*B*_1_	*A*_1_	*A*_2_	*G*	*F*	*p*
	30	40	20	10	20	0.1	40	35	15	20	50	0.9

### 5.2 Comparative analysis of evolutionary pathways

**Figure 2:**
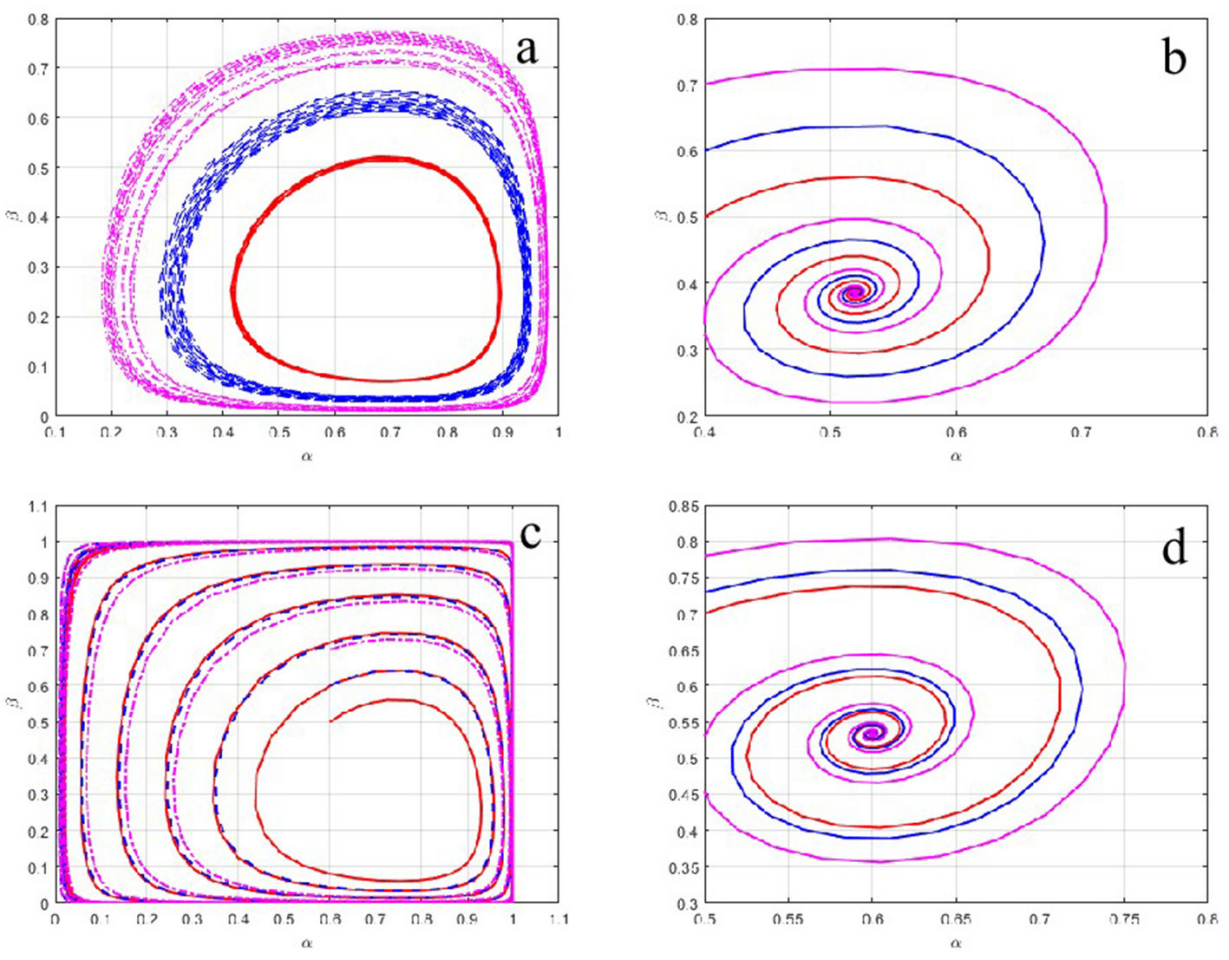
**(a)** Evolutionary pathways of occupational health risk management strategies for polluting enterprises and local governments under SRSP mechanism. **(b)** Evolutionary pathways of occupational health risk management strategies for polluting enterprises and local governments under SRDP mechanism. **(c)** Evolutionary pathways of occupational health risk management strategies for polluting enterprises and local governments under DRSP mechanism. **(d)** Evolutionary pathways of occupational health risk management strategies for polluting enterprises and local governments under DRDP mechanism.

The evolutionary trajectories of the system under the four regulatory configurations are illustrated in Figure 2.

As depicted in Figures 2a, c, under the SRSP and DRSP mechanisms, the evolutionary paths form closed circular orbits. The system fails to converge to a stable equilibrium point, instead exhibiting persistent periodic oscillations. This mirrors the empirical reality of campaign-style enforcement. Compliance fluctuates cyclically with regulatory pressure. In contrast, spiral trajectories are displayed in Figures 2b, d for mechanisms with dynamic penalties (SRDP and DRDP). These trajectories asymptotically converge to the stable equilibrium point *M*(α^*^, β^*^).

A comparative assessment reveals that the DRDP mechanism achieves convergence most rapidly and stabilizes at a higher probability level for both active governance (α) and strict supervision (β). This superiority stems from the dual-feedback loop of DRDP, which embodies the core characteristic of adaptive governance. Incentives are calibrated continuously in response to behavioral changes. Consequently, the cat-and-mouse dynamics inherent in static regulation are corrected.

### 5.3 Sensitivity analysis of key parameters

Based on the DRDP framework, the influence of variations in profit and cost differentials, reward and penalty intensities is examined.

#### 5.3.1 Impact of profit and cost differentials

**Figure 3:**
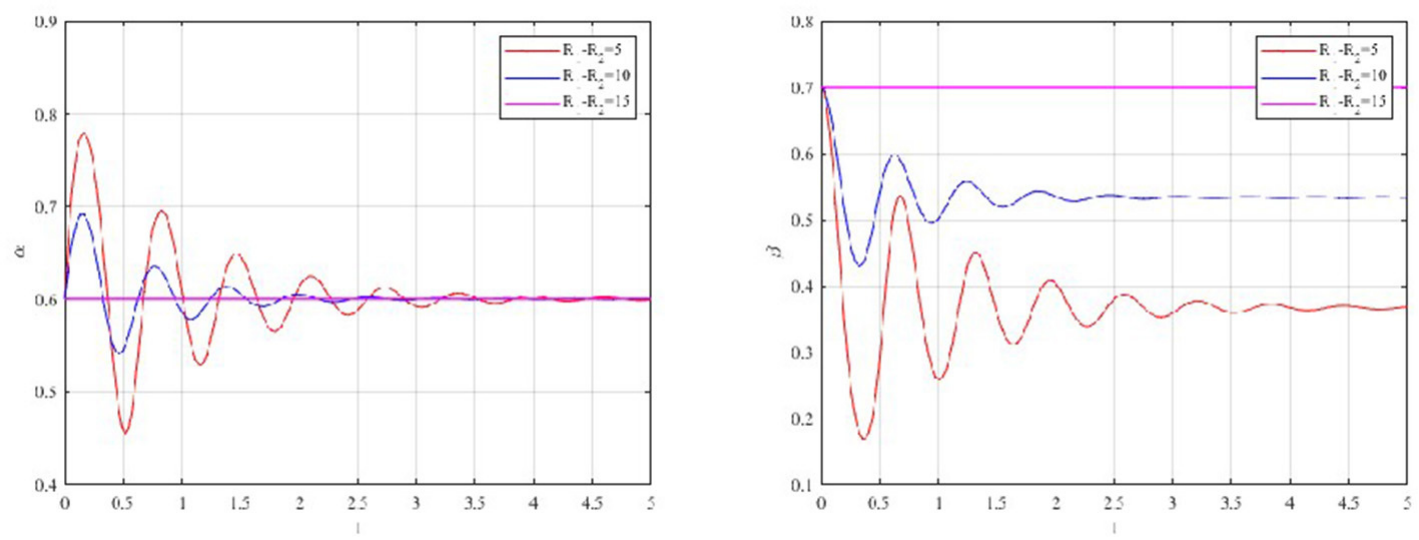
Impact of benefit differential on occupational health management strategy.

**Figure 4:**
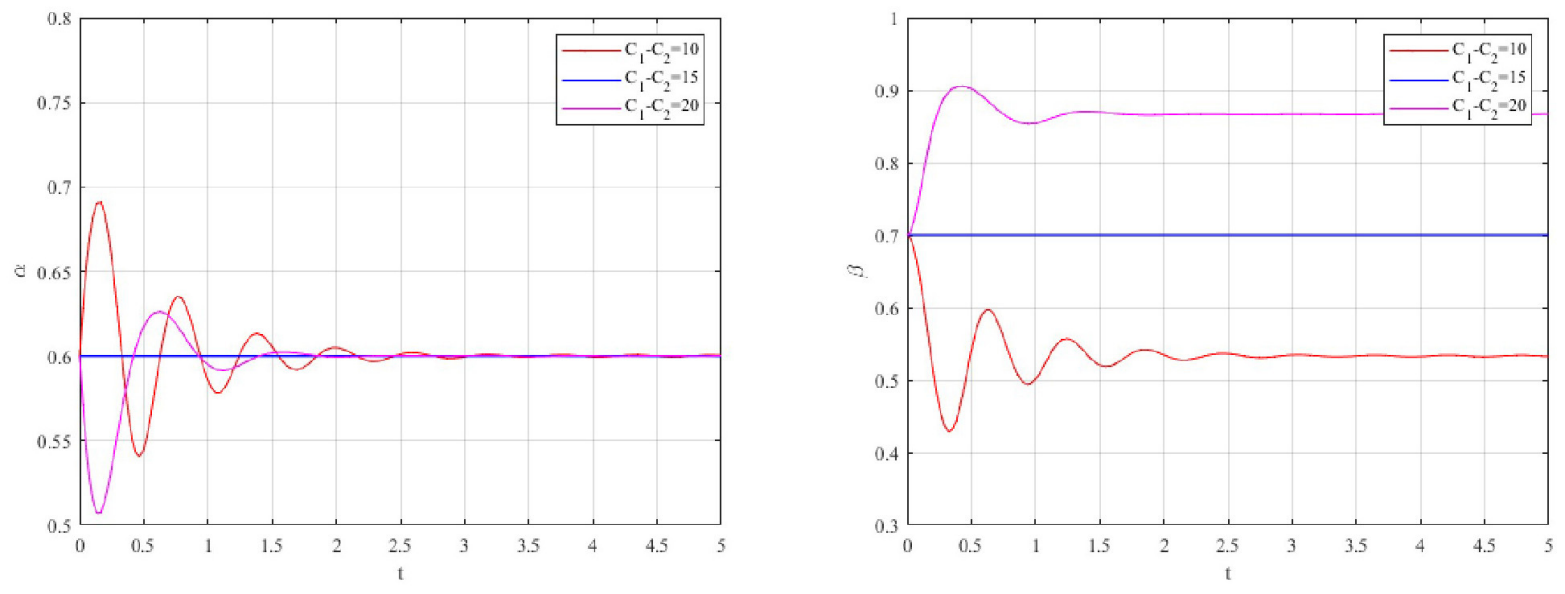
Impact of cost differential on occupational health management strategy.

The sensitivity of evolutionary paths to changes in the benefit differential (*R*_1_−*R*_2_) and cost differential (*C*_1_−*C*_2_) is displayed in Figures 3, 4.

As shown in Figures 3, 4, moderate fluctuations in production costs or revenues have a relatively negligible impact on the convergence speed, suggesting that occupational health prevention is more sensitive to regulatory design than to short-term economic variation. This counter-intuitive finding reflects the myopic cognitive bias inherent in corporate decision-making (69). Since the benefits of occupational health (e.g., accident prevention, reputation) are delayed and probabilistic. Conversely, costs are immediate and certain. Therefore, firms are less sensitive to marginal changes in operational parameters. Strong external constraints are required to alter behavior.

#### 5.3.2 Impact of reward intensity

**Figure 5:**
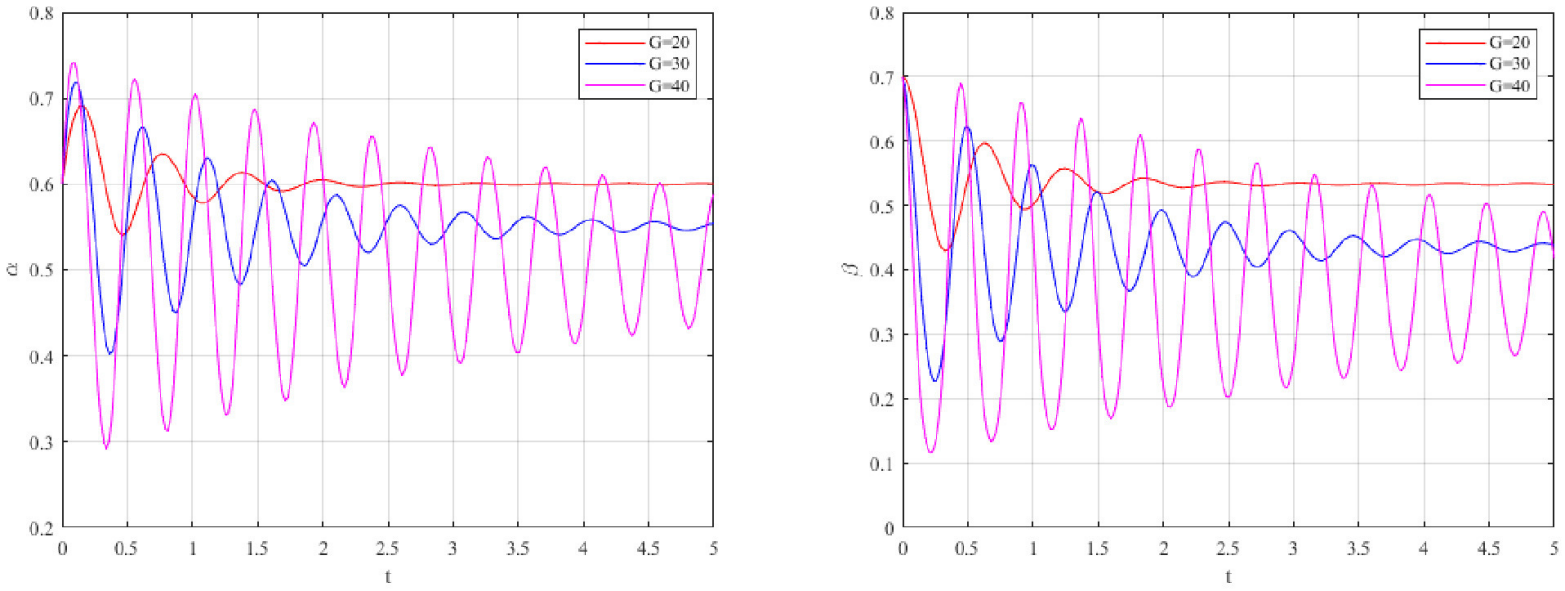
Impact of reward on occupational health management strategies.

The system's response to varying maximum reward caps (*G*_*m*_) is illustrated in Figure 5.

A decreasing trend is observed for both the probability of active governance (α) and strict supervision (β) as reward intensity increases, implying diminishing returns of subsidy-based approaches for occupational health protection. High subsidies initially stimulate enterprise compliance. However, the government's willingness to regulate is rapidly suppressed. This reveals a fiscal paradox (13). Excessive rewards impose an unsustainable fiscal burden on local regulators. As compliance rates rise, the aggregate subsidy payout increases significantly. This forces the government to relax supervision to cut enforcement costs(*C*_*g*_). Consequently, enterprises anticipate reduced oversight. A “wait-and-see” approach is adopted. The system stabilizes at a lower-than-optimal level.

#### 5.3.3 Impact of penalty severity

**Figure 6:**
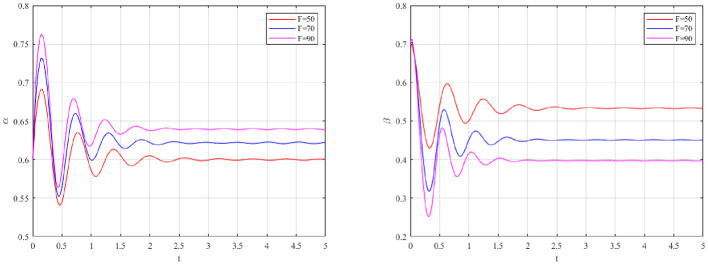
Impact of punishment on occupational health management strategies.

The evolutionary impact of increasing the maximum penalty ceiling (*F*_*m*_) is depicted in Figure 6.

Unlike rewards, increasing penalty severity is shown to significantly elevate the equilibrium probability of Active Governance (α), thereby strengthening occupational health prevention even under reduced inspection intensity. However, a gradual decrease in Strict Supervision (β) is also observed. High compliance reduces the necessity for frequent inspections. This asymmetry validates the loss aversion principle derived from prospect theory (70, 71). Firms are significantly more sensitive to the threat of penalties than to the allure of subsidies. Furthermore, high penalties act as a credible deterrent. High compliance is maintained even when inspection frequency is reduced. A cost-effective high compliance, moderate supervision equilibrium is thus achieved.

#### 5.3.4 Impact of monitoring probability

**Figure 7:**
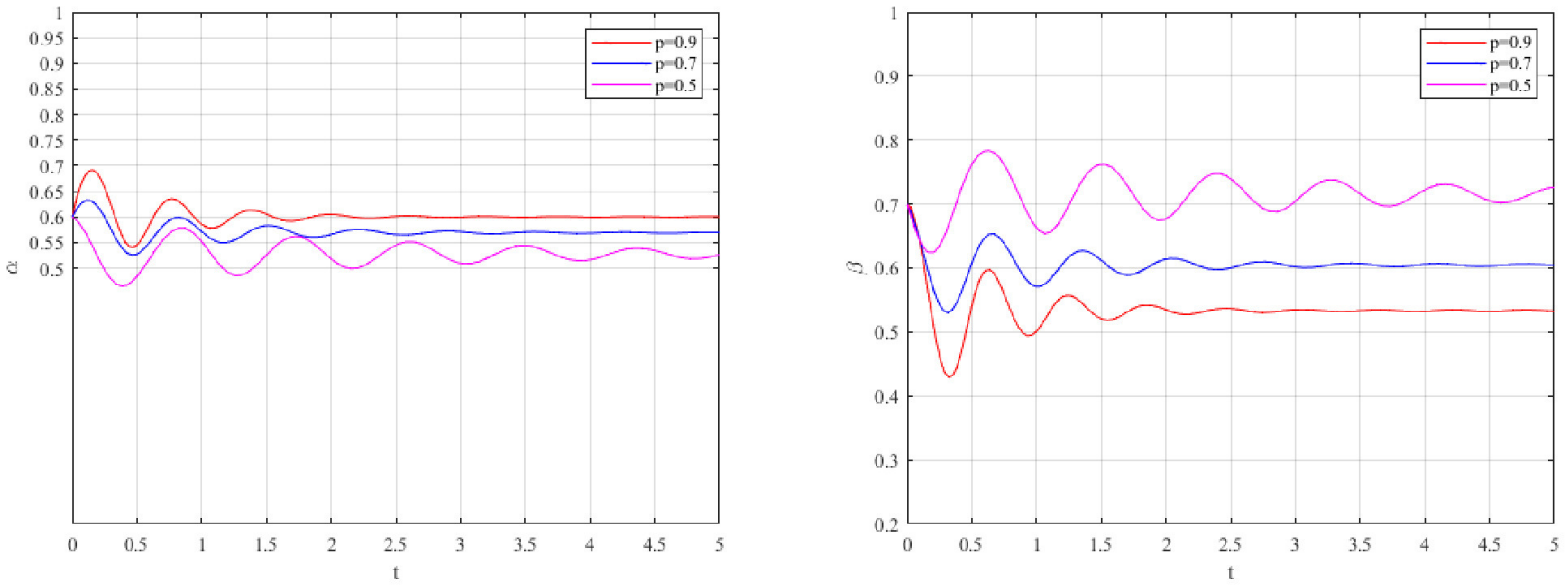
Impact of monitoring probability on occupational health management strategies.

The evolutionary impact of monitoring probability (p) is shown in Figure 7.

As shown in Figure 7, the system was highly sensitive to changes in the monitoring probability p. As p increased, α converged to a higher stable level, which indicates that stronger monitoring effectiveness enhanced the expected penalty on passive governance and improved the relative attractiveness of active governance. In contrast, β converged to a lower stable level, suggesting that effective detection reduced the need for sustained high-intensity supervision. When p remained low, the stable level of α was lower, whereas the government had to maintain a higher level of strict supervision to offset weak observability. Overall, the results show that the effectiveness of the regulatory mechanism depends not only on incentive intensity, but also on the monitoring probability.

## 6 Discussion

The evolutionary stability analysis presented in this study suggests that dynamic reward–penalty mechanism is likely a prerequisite for effective co-governance in occupational health protection. Under static regulatory signals (SRSP), the strategies of governments and enterprises exhibit persistent cyclical oscillations rather than convergence, undermining sustained occupational health protection. This pattern aligns with empirical observations of campaign-style enforcement, which weakens occupational health prevention by encouraging short-term compliance rather than sustained risk reduction (72–74). Static mechanisms appear insufficient to lock firms into long-term compliance paths, and therefore fail to secure stable occupational health outcomes (75, 76). Unlike previous frameworks that largely treat environmental metrics and occupational health as separate domains (77, 78), the proposed DRDP mechanism integrates occupational health governance into the regulatory game as a compliance outcome with public-health-relevant implications. By endogenizing occupational health risks into the penalty variable and allowing passive governance to be detected under strict supervision with probability p through observable compliance signals, the model encourages firms to internalize the co-produced risks of pollution and worker safety, thereby potentially improving worker protection and reducing accident-related social losses. This approach offers a theoretical basis for strengthening the linkage between external emissions control and internal workplace safety (79, 80), particularly when occupational health indicators are incorporated into regulatory inspections, compliance evaluations, or disclosure systems.

Furthermore, the sensitivity analysis reveals a critical asymmetry in behavioral responsiveness that enriches the theory of policy instrument design. The simulation indicates that increasing penalty intensity exerts significantly stronger leverage on corporate compliance than increasing rewards. This finding provides a behavioral nuance to the traditional subsidy-driven view, with important implications for occupational health governance (81). Instead, the results resonate with Prospect Theory and the principle of loss aversion (82). Bounded rationality leads firms to perceive regulatory penalties as existential threats, making penalty-based mechanisms more effective than reward-only approaches in promoting active occupational health governance (83). This explains why robust penalties often serve as the necessary baseline for regulatory credibility, dynamic reward mechanism may overlook (84, 85). Importantly, the sensitivity analysis of monitoring probability indicates that stronger observability further reinforces this effect, since improved monitoring capacity can strengthen enterprise self-discipline (20, 29).

Finally, the study sheds light on the fiscal paradox associated with high-reward policies. The results indicate that excessive rewards may inadvertently suppress the government's capacity to enforce strict regulation over time, thereby weakening sustained occupational health protection. High subsidies create a substantial fiscal burden for local regulators, potentially forcing a relaxation of oversight to reduce costs. This observation differs from the classical assumption where the government is viewed as a benevolent planner with unlimited resources (86). In the context of local implementation under fiscal constraints, high penalties coupled with moderate rewards structure appear to be more robust for maintaining long-term occupational health protection. It ensures regulatory resilience by minimizing fiscal pressure and preventing a form of passive regulatory capture where the regulator lacks the resources to police the standards they subsidize (87, 88).

## 7 Conclusions and implications

This study constructs an evolutionary game framework to address the governance gap between environmental performance and occupational health. By comparing four regulatory configurations (SRSP, SRDP, DRSP, and DRDP), we uncover the micro-foundations of firm compliance and regulatory effectiveness. In this study, the dynamic aspect of regulation is operationalized specifically through state-dependent reward-penalty adjustment within an evolutionary interaction. The core findings are as follows:

First, state-dependent incentive adjustment is a prerequisite for stability. Under static mechanisms (SRSP and SRDP), the absence of feedback loops leads to cyclical campaign-style enforcement, where firms temporarily comply during inspections but relapse thereafter. In contrast, the Dynamic Reward and Dynamic Penalty (DRDP) mechanism successfully drives the system toward an asymptotically stable equilibrium, achieving the highest level of synergistic governance.

Second, penalties outweigh rewards in driving compliance. Our simulation reveals a distinct asymmetry: firms are significantly more sensitive to penalty intensity than to reward magnitude. This validates the loss aversion principle in corporate decision-making, suggesting that while subsidies are welcomed, they are insufficient to overcome the myopic cognitive bias associated with the delayed benefits of occupational health investments. More importantly, strict supervision can be observed as a condition of imperfect but improved detection, which allows the effectiveness of state-dependent incentive adjustment to depend not only on incentive intensity, but also on the monitoring probability that passive governance can actually be observed.

Third, the fiscal paradox constrains high-reward policies. We identify a critical trade-off: excessive rewards increase the fiscal burden on local governments, inadvertently reducing their capacity and willingness to enforce strict regulation. Consequently, a high Penalty, moderate Reward structure emerges as the robust optimal design, preventing the passive regulatory capture caused by fiscal exhaustion. This result further suggests that effective occupational health governance should rely on a balanced combination of credible penalties, moderate rewards, and workable monitoring capacity.

Together, these findings indicate that effective occupational health governance requires state-dependent, penalty-centered regulatory arrangements with public-health-relevant implications. Based on these findings, we propose a transition from fragmented regulation to integrated smart governance. First of all, establish an integrated Green-Health dual-credit system. Policymakers should dismantle the data silos between environmental protection and emergency management departments. We recommend a unified credit rating system where occupational safety records are embedded into existing environmental profiles. A red card in worker safety should automatically trigger restrictions in environmental permitting and financing, enforcing the principle that green development must also be safe development. Hence, the influence of environmental regulation on occupational health governance can be transmitted through institutional channels such as compliance evaluation, regulatory inspection, and information disclosure. Then, implement a dynamic circuit breaker mechanism. To avoid the rigidity of static regulation, enforcement intensity should be algorithmically linked to corporate credit ratings. For green enterprises, inspection frequency should be reduced to lower compliance costs. However, for yellow or red enterprises, a dynamic penalty multiplier should be automatically triggered: escalating fines and inspection frequency until the firm returns to compliance. This creates a credible threat that corrects firms' intertemporal valuation errors. Last but not least, shift from fiscal subsidies to market-based insurance. To resolve the fiscal paradox, governments should reduce direct subsidies and instead leverage market instruments. Specifically, we advocate for the mandatory adoption of safety liability insurance with floating rates. By linking insurance premiums to safety performance, the financial incentive for risk prevention is internalized by the market (insurers) rather than subsidized by the public purse (government), ensuring the long-term sustainability of the regulatory regime.

While this study offers a novel theoretical framework, limitations remain. In this study, while only passive governance observability is modeled, future work may distinguish active and passive signals or incorporate third-party audits. In addition, the public health relevance of the model should be interpreted with appropriate caution. In the present framework, occupational health consequences are represented mainly through accident-related losses, regulatory costs, and reputational effects, rather than explicit epidemiological outcomes. Further work may also incorporate explicit health outcomes, such as disease burden, exposure reduction, or long-term health trajectories based on this research. Such extensions would deepen understanding of how adaptive regulatory mechanisms perform under more complex institutional conditions.

## Data Availability

The original contributions presented in the study are included in the article/supplementary material, further inquiries can be directed to the corresponding author.
